# Abdominal Muscle Activity during Mechanical Ventilation Increases Lung Injury in Severe Acute Respiratory Distress Syndrome

**DOI:** 10.1371/journal.pone.0145694

**Published:** 2016-01-08

**Authors:** Xianming Zhang, Weiliang Wu, Yongcheng Zhu, Ying Jiang, Juan Du, Rongchang Chen

**Affiliations:** 1 First Affiliated Hospital of Guangyang Medical University, Guangyang, Guizhou, China; 2 Respiratory Mechanics Lab, State Key Laboratory of Respiratory Disease, Guangzhou Institute of Respiratory Disease, First Affiliated Hospital of Guangzhou Medical University, Guangzhou, Guangdong, China; Chinese Academy of Sciences, CHINA

## Abstract

**Objective:**

It has proved that muscle paralysis was more protective for injured lung in severe acute respiratory distress syndrome (ARDS), but the precise mechanism is not clear. The purpose of this study was to test the hypothesis that abdominal muscle activity during mechanically ventilation increases lung injury in severe ARDS.

**Methods:**

Eighteen male Beagles were studied under mechanical ventilation with anesthesia. Severe ARDS was induced by repetitive oleic acid infusion. After lung injury, Beagles were randomly assigned into spontaneous breathing group (BIPAP_SB_) and abdominal muscle paralysis group (BIPAP_AP_). All groups were ventilated with BIPAP model for 8h, and the high pressure titrated to reached a tidal volume of 6ml/kg, the low pressure was set at 10 cmH_2_O, with I:E ratio 1:1, and respiratory rate adjusted to a PaCO_2_ of 35–60 mmHg. Six Beagles without ventilator support comprised the control group. Respiratory variables, end-expiratory volume (EELV) and gas exchange were assessed during mechanical ventilation. The levels of Interleukin (IL)-6, IL-8 in lung tissue and plasma were measured by qRT-PCR and ELISA respectively. Lung injury scores were determined at end of the experiment.

**Results:**

For the comparable ventilator setting, as compared with BIPAP_SB_ group, the BIPAP_AP_ group presented higher EELV (427±47 *vs*. 366±38 ml) and oxygenation index (293±36 *vs*. 226±31 mmHg), lower levels of IL-6(216.6±48.0 *vs*. 297.5±71.2 pg/ml) and IL-8(246.8±78.2 *vs*. 357.5±69.3 pg/ml) in plasma, and lower express levels of IL-6 mRNA (15.0±3.8 *vs*. 21.2±3.7) and IL-8 mRNA (18.9±6.8 *vs*. 29.5±7.9) in lung tissues. In addition, less lung histopathology injury were revealed in the BIPAP_AP_ group (22.5±2.0 *vs*. 25.2±2.1).

**Conclusion:**

Abdominal muscle activity during mechanically ventilation is one of the injurious factors in severe ARDS, so abdominal muscle paralysis might be an effective strategy to minimize ventilator-induce lung injury.

## Introduction

Mechanical ventilation is the main therapy for patients suffering from ARDS, which will improve oxygenation, reduce the work of breathing and prevent muscle fatigue. However, mechanical ventilation itself might aggravate lung injury [1,2]. Although there is a widely use of the mechanical ventilation strategies such as low tidal volume, lower airway plateau pressure, optimal positive end-expiratory pressure (PEEP), permissive hypercapnia, patients with ARDS still has a higher morbidity and mortality in the past two decades [3].

Invasive mechanical ventilation for patients with ARDS include preserving spontaneous breathing (SB)and controlled mechanical ventilation (CMV). Whether SB should be preserved has been debated for many years. Animal experiments [4,5] and clinical studies [6] have reported that CMV may increase alveolar collapse and inhomogeneity of pulmonary parenchyma, and thus induce further lung injury. SB during mechanical ventilation results in better aeration and less atelectasis in lung dependent zones, and less hyperinflation in nondependent lung zones in ARDS [7]. Some researchers have claimed that, even in the most severe ARDS, preserving SB activity was associated with beneficial effects in pulmonary function, speeding of weaning, and discharge from the ICU [8]. However, in a recent multicenter trial, Papazian et al [9] found that, in patients with severe ARDS, muscle paralysis was associated with a lower adjusted 90 days morbidity than patients that received placebo.Yoshida et al [10] also found that in animal with severe ARDS, muscle paralysis might be more protective for injured lung, and SB could worsen lung injury. However, the precise mechanism is unclear.

Until now, it is unknown whether the activity of abdominal muscles has any impact on VALI. Caironi et al [11] has recently proved that the amount of lung cyclically recruitment and decruitment, but not lung stress and strain, leads to the increase of mortality in ARDS patients. Expiration in mechanical ventilation, as well as in normal breathing, is a passive phenomenon produced due to the lung elastic recoil forces. However, in the presence of increased respiratory driving, expiratory muscles, especially the abdominal muscles may actively participate in breathing and increase intra-abdominal pressure. It has been proved that the increase of intra-abdominal pressure even 10 cmH_2_O may have injurious impacts on organ functions [12] and aggravate lung damage [13]. Therefore, we hypothesized that abdominal muscle activity during mechanically ventilation increases lung injury in severe acute respiratory distress syndrome. In this study we explored the hypothesis.

## Materials and Methods

This study was approved by the ethics committee of Guangzhou medical university. Experimental animals obtained from the Kangda laboratory animals Science & Technology Company of Gaoyao city and the care and treatment of the animals were in compliance with the university standard.

### Animal Preparation

Eighteen adult male Beagles (10.5–14.3 kg) were recruited in this experiment. After intramuscular injection of 3% pentobarbital sodium (30 mg/kg), the Beagles were studied in the supine position and anesthetize with Propofol by continuous infusion (0.16–0.5 mg/ kg/ h). Paralysis was achieved with pancuronium(bolus = 0.16 mg /kg, followed by 0.08 mg /kg/ h). After orotracheal intubation with an 8.0 mm ID cuff tube, lungs were ventilated with the ventilator EVITA 4 (Dräger Medical AG, Lübeck, Germany). Volume-controlled model with a tidal volume (VT) of 10 ml/kg, PEEP 5 cm H_2_O, I:E ratio 1:1, FiO_2_ 1.0 and the respiratory rate(RR)was adjusted to maintain PaCO_2_ between 35 and 45 mmHg. Intravenous fluid (lactated Ringer^’^s; 6 ml /kg/ h) were administrated to maintain the mean arterial blood pressure more than 70 mmHg. The right jugular vein and femoral artery were catheterized and connected to PiCCO system for measure of core temperature, mean arterial blood pressure(MPA) and obtain artery blood sample for gas analysis. A multipair esophageal electrode-balloon combined catheter was put into the esophagus, and airway occlusion technique was used to check the proper position [14]. Airway pressure (Paw), esophageal pressure (Peso) and intragastric pressure (Pgas) from a side tap was connected to the end tracheal. A MLT300L respiratory flow head was used to measure airflow, and integrated airflow to obtain tidal volume. Signal of Paw, Peso, Pgas, airflow, diaphragmatic esophageal surface electromyography (EMGdi) and abdominal muscles surface electromyography (EMGab) were recorded by powerlab 16/30 SP and chart 7.2 software on a Mac book Computer. Body temperature was constant throughout the whole experiment at 37°C with a heating pad.

### Experimental Protocol

**Fig 1:**
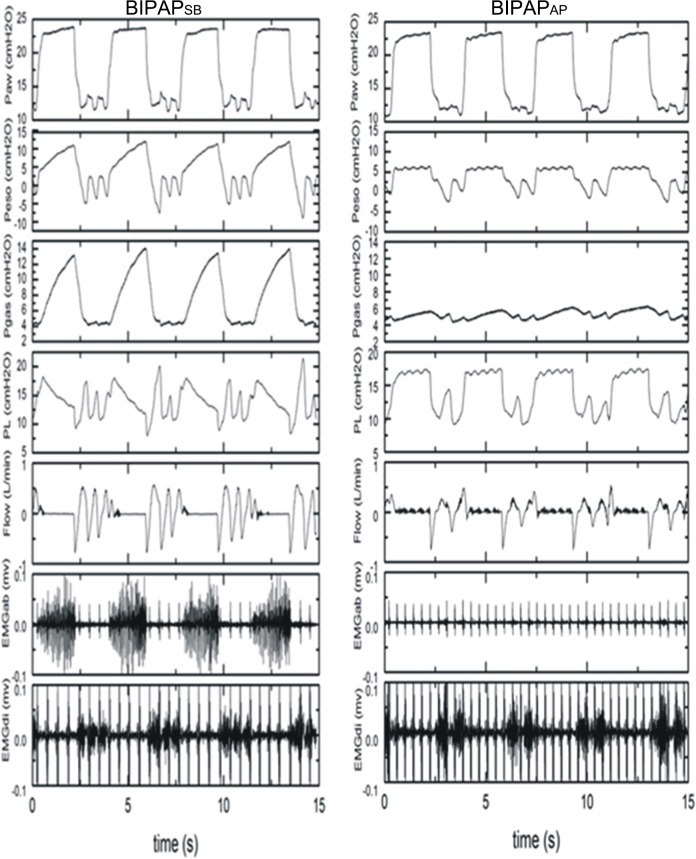
Representative respiratory tracings. Representative respiratory tracings of airway pressure(Paw), esophageal pressure (Pes), intragastric pressure (Pgas),transpumonary pressure (PL),Airflow、abdominal muscles surface electromyography (EMGab)and diaphragmatic esophageal surface electromyography (EMGdi) in BIPAP_SB_,BIPAP_AP_ group in representative animals. BIPAP_SB_ = biphasic positive airway pressure with SB; BIPAP_AP_ = biphasic positive airway pressure with abdominal muscles paralysis.

After obtaining baseline measurements and 30 minute stabilization, lung injury was induced by the total dose of 0.30 ml/kg purified oleic acid. If necessary, additional injection oleic acid (0.2 ml each time) was given until PaO_2_/FiO_2_ were below 100 mmHg. A stable severe ARDS model was established when the PaO_2_/FiO_2_ value remain less than 100 mmHg within 30 min [15,16,17]. After obtaining the measurements at injury, ventilator mode switched to BIPAP mode and the animals were randomly divided into SB group (BIPAP_SB_ group) and abdominal muscle paralysis group(BIPAP_AP_ group). After muscles paralysis, the P_high_ titrated to achieve a VT of 6 ml/kg, P_low_ was set at 10 cm H_2_O, with FiO_2_ 1.0, I:E was fixed at 1:1 to minimize changes in mean Paw. The mandatory RR adjusted a PaCO_2_ of 35 to 60 mmHg. In BIPAP_SB_ group, in order to recover SB, stopped the injection of pancuronium and gradually reduced the dose of Propofol. According to previous studies, in order to ensure a strong effort of unsupported SB during BIPAP, the mandatory RR was adjusted to maintain the percentage of minute ventilation (MV) of unsupported SB to total MV >50% (Fig 1).Other ventilator settings remained the same as the above setting. SB was monitored by online registration of the Peso signal. The amount of SB was quantified by measuring minute volumes before and after neuromuscular blockade. In the BIPAP_AP_ group, the abdominal muscles paralysis method was described as Warner DO[18], a flexible epidural catheter was placed percutaneous through the second coccygeal vertebra and advanced until the tip closed to the L4 or L5 vertebra in the epidural space as confirmed by visual observation and autopsy. Through the epidural catheter, 2% Lidocaine was injected in 0.5 ml increment until the EMGab was abolished and followed by continuous infusion of Ropivacaine Hydrochloride 1-2ml/h. All the other ventilator setting were totally same with BIPAP_SB_ group.

### Respiratory Mechanics

All variables were recorded continuously. Mean Paw for the BIPAP mode can be calculated as follows [19,20]: (P_high_ × T_high_ + P_low_ × T_low_) / (T_high_+ T_low_), where the T_high_ is the length of time for P_high_; T_low_: length of time during P_low_. If the ratio of T_high_: T_low_ is fixed at 1:1, then the mean Paw could be kept constant when we changed the RR.Using above method adjust ventilator, we could maintain the level of mean Paw comparable in our study. The transpulmonary pressure (P_L_) was calculated as Paw–Peso. Peak Paw were recorded. Peso swings as the frequency per minute of each type of breathing cycle was used to calculate the total RR (S1 Fig). The product of inspiratory Peso vs. time (PTP) were determined.

### EELV

EELV at PEEP or P_low_ was measured by simplified helium dilution method. A flexible tube inserted between endotracheal tube and the circuit Y and clamped during an end-expiratory pause at PEEP. Anesthesia balloon filled with1.5 L known gas mixture of Helium (13.4%) in oxyge was then connected to the tube. After releasing the clamp, 10 tidal volumes compressing were performed rhythmically to dilute the helium gas mixture with the gas contained in Beagles lungs. Concentration of the helium in the bag was then measured with the helium analyzer (c-square company, USA), the following formula was used to computed the EELV (ml) = $\frac{\text{Vi}\times \text{Ci}}{\text{Cf}}$ - Vi, where Vi: initial gas volume in the bag; Ci: initial helium concentration; and Cf: the final helium concentration [21].

### End-tidal CO_2_ (ETCO_2_)

A pressure differential pneumotachometer was used to measure end-tidal CO_2_ (ETCO_2_). The alveolar dead space fraction (VD/VT) was calculated by [22]: VD/VT = $\frac{\text{PaCO}2-\text{ETCO}2}{\text{PaCO}2}$

### Levels of Inflammatory Mediators

Plasma samples was collected at baseline, lung injury and at the end of 8h MV. After centrifuged at 3,000 rpm for 15 min,Supernatant aliquots were frozen at -80°C for analysis. Plasma levels of IL-6 and IL-8 were measured using an ELISA kit for dogs (Genequick, Guangzhou, China) according to manufacturer protocol. Expression levels of IL-6 and IL-8 mRNA in lung tissues were measured by qRT-PCR as previously described [20]. GAPDH primers were used as an internal control. The sense and antisense of the primers (5’-3’) used for IL-6 and IL-8 were:

IL-6 F: TGACCACTCCTGACCCAACC, R: TCCAGACTCCGCAGGATGAG;
IL8F: ACTTCCAAGCTGGCTGTTGC, R: CTGGCATCGAAGTTCTGAACTG.

### Histopathological Examination

After 8 hours of ventilation, all Beagles were euthanized by intravenous injection of potassium chloride, and lung tissues were harvested (S2 Fig). Samples were obtained separately from the upper lobe, middle lobe and ventral, lateral and dorsal sections of the right lower lobe. Samples were fixed in 10% formalin and were stained by HE for histological analysis. All sections were examined by the same pathologist and were evaluated by the following lung injury scores system [4,23]: 0, minimal changes; 1, mild; 2, moderate; 3, severe; 4, maximal changes. For each slide including the following criteria: congestion, alveolar and interstitial edema, granulocytes, lymphocytes and erythrocytes infiltration, fibrinous exudates and micro thrombi. The sum of pathological score was calculated by adding the cumulative lung injury sub-scores (maximal value is 44).

### Statistical Analysis

All date were represented as means ± SDs. Normal distribution of the data were assessed by the Kolmogorov–Smirnov test. Comparison of data between two experimental groups with each other was performed using the unpaired t test or Mann-Whitney tests as appropriate. Comparison of the continuous date within the same group before and after the experiments were evaluated by Paired *t* tests. ANOVA or Kruskal-Wallis test were applied for multiple-group comparisons as appropriate. Effects of time and group differences on respiratory variables were evaluated by Repeated Measures Two-way ANOVA. The LSD post-hoc test was used as appropriate. IBM SPSS Statistics 21 was used for statistical analyses. Differences were considered to be statistically significant if P was less than 0.05.

## Results

Fig 1 Shows tracing records of Paw, Pes, Pgas, P_L_, Airflow, EMGab and EMGdi for the two groups in representative animals. There were no difference in the value of mean Paw, and the only difference was the absence of abdominal muscle activity in BIPAP_AP_ group. SB occurred rarely at P_high_ in the experimental groups. The average percentage of minute ventilation of unassisted SB relative to total minute ventilation in the BIPAP_SB_ group was above 50%.After abdominal muscle paralysis, the percentage decreased from 50%-100% to 10% -50%.

**Fig 2:**
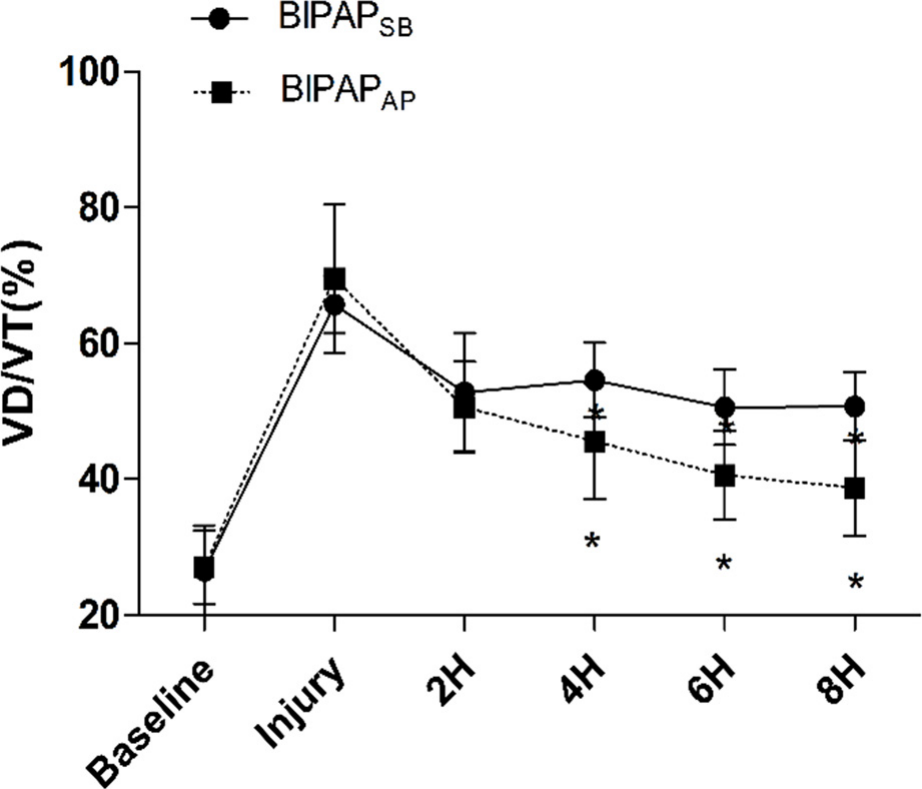
Time course of the VD/VT. Time course of the dead space volume to tidal volume (VD/VT) ratio in experimental groups (n = 6 per group). BIPAP_SB_ = biphasic positive airway pressure with SB; BIPAP_AP_ = biphasic positive airway pressure with abdominal muscles paralysis; SB = spontaneous breathing; *P < 0.05, vs. other groups.

**Fig 3:**
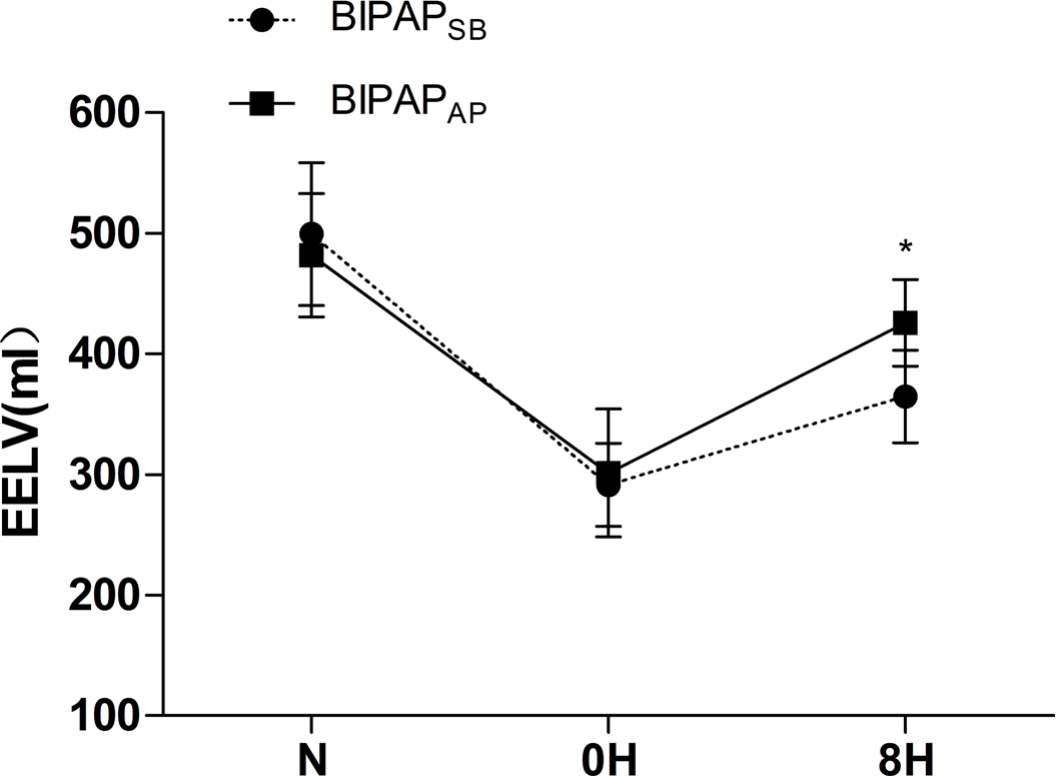
Time course of the EELV. Time course of the end- expiratory lung volume (EELV) in experimental groups (n = 6 per group). BIPAP_SB_ = biphasic positive airway pressure with SB; BIPAP_AP_ = biphasic positive airway pressure with abdominal muscles paralysis; SB = spontaneous breathing; *P < 0.05, vs. other groups.

As shown in Table 1, at baseline, there are no differences in HR、MAP between the groups during the entire experiment. There were also comparable mean Paw between the experimental groups. The PaCO_2_ level was less than 60 mmHg in all of the animals. Due to activity of the diaphragm and abdominal muscles, the BIPAP_SB_ group presented higher swing of Pes, Pgas and peak P_L_ than BIPAP_AP_ group. After abdominal muscle paralysis, BIPAP_AP_ group presented lower swing of Peso, Pgas, peak P_L_, more even P_L_ and longer time on P_high_ (S1 Table). Moreover, the BIPAP_AP_ group resulted in a higher EELV (427±47 ml) compared with the BIPAP_SB_ group (366±38 ml) (Fig 2). Meanwhile, BIPAP_AP_ group showed a lower VD/VT than BIPAP_SB_ group (Fig 3). BIPAP_AP_ group showed a trend toward improving PaO_2_/FiO_2_, but not in BIPAP_SB_ group. The difference in PaO_2_/FiO_2_ between two groups was statistically significant after 2h MV (*p* = 0.025). PTP decreased gradually from BIPAP_SB_ group to BIPAP_AP_ group.

**Table 1:** Respiratory Measurements.

Variables	Group (n = 6)	Before ARDS	After Induction of ARDS					Group Effect	Time*Group Effect
			Injury	2 h	4 h	6 h	8 h		
**HR (beats/min)**	**BIPAP**_**AP**_	134±16	131±23	130±11	124±9	119±22	133±12	0.292	0.938
	**BIPAP**_**SB**_	142±11	126±22	130±10	115±15	120±17	126±12		
**MAP(mmHg)**	**BIPAP**_**AP**_	111±6	125±6	116±10	112±13	117±13	106±13	0.925	0.238
	**BIPAP**_**SB**_	109±11	123±10	108±10	109±16	114±14	122±18		
**PaO**_**2**_**/FiO**_**2**_**(mmHg)**	**BIPAP**_**AP**_	407±33	118±16	154±34*	205±29*	269±52*	293±36*	0.025	0.004
	**BIPAP**_**SB**_	421±34	117±19	126±19	170±27	197±32	226±31		
**PaCO**_**2**_**(mmHg)**	**BIPAP**_**AP**_	42.3±3.5	56.4±18.6	58.6±12.3	58.2±3.9	58.9±9.1	58.9±10.6	0.793	0.889
	**BIPAP**_**SB**_	45.9±7.8	54.7±10.7	52.5±7.8	54.2±7.8	53.6±5.9	57.6±15.3		
**Total RR**	**BIPAP**_**AP**_	21±3	36±7	37±6	35±10	36±6	35±9	0.110	0.956
**(breaths/min)**	**BIPAP**_**SB**_	22±7	35±8	38±12	37±11	38±9	37±10		
**VT**_**ave**_**(ml/kg)**	**BIPAP**_**AP**_	9.8±0.3	6.1±0.9	6.2±1.0	6.1±0.9	6.3±0.9	6.1±0.7	0.167	0.472
	**BIPAP**_**SB**_	10.1±0.3	6.5±1.6	6.6±1.2	6.4±1.4	6.4±1.5	6.5±1.8		
**MV**_**tot**_**(L/min)**	**BIPAP**_**AP**_	2.8±1.1	3.1±1.7	3.4±0.5	3.3±1.3	3.2±0.9	3.3±1.5	0.343	0.623
	**BIPAP**_**SB**_	2.8±0.9	3.3±1.8	3.5±0.8	3.2±1.1	3.3±1.1	3.2±1.2		
**P**_**plat**_ **(cmH**_**2**_**O)**	**BIPAP**_**AP**_	9.8±1.2	22.3±1.4	21.7±1.6	22.6±1.8	21.7±1.7	22.3±1.2	0571	0.216
	**BIPAP**_**SB**_	9.5±1.8	21.9±1.2	21.4±1.9	22.7±1.7	22.8±1.6	22.1±1.5		
**Mean Paw (cmH2O)**	**BIPAP**_**AP**_	7.7±0.7	17.6±1.2	17.9±0.8	17.1±1.3	17.7±1.2	17.3±1.2	0.516	0.216
	**BIPAP**_**SB**_	7.6±0.6	17.1±1.1	17.8±1.2	18.0±1.2	17.8±1.4	17.2±1.1		
**Peak P**_**L**_ **(cmH**_**2**_**O)**	**BIPAP**_**AP**_	7.2±1.4	17.2±1.4*	17.3±1.1*	17.7±1.3*	17.3±1.5*	17.3±1.9*	0.015	0.393
	**BIPAP**_**SB**_	6.8±1.6	19.9±1.0	20.1±1.9	21.2±1.7	20.9±0.6	21.5±1.4		
**ΔPes(cm H**_**2**_**O)**	**BIPAP**_**AP**_	—	4.3±0.5	8.5±0.7*	7.8±0.7*	8.5±0.8 *	7.8±1.0 *	0.018	0.520
	**BIPAP**_**SB**_	—	4.5±0.6	14.7±1.5	15.5±2.0	15.2±1.9	14.0±2.2		
**Pgas(cm H**_**2**_**O)**	**BIPAP**_**AP**_	—	5.4±1.3	5.9±0.6*	6.1±0.9*	5.1±0.8*	5.7±1.3*	0.001	0.582
	**BIPAP**_**SB**_	—	5.6±1.1	12.3±1.0	13.5±2.0	12.1±2.6	11.8±2.0		
**PTP ml**	**BIPAP**_**AP**_	—	—	43±19*	39±19*	54±22*	45±26*	0.025	0.438
	**BIPAP**_**SB**_	—	—	97±33	88±45	96±37	88±39		

Values are means ± SD. ARDS = acute respiratory distress syndrome; BIPAP_SB_ = biphasic positive airway pressure with SB; BIPAP_AP_ = biphasic positive airway pressure with abdominal muscles paralysis; SB = spontaneous breathing; MV = minute ventilation; PaCO_2_ = partial pressure of carbon dioxide; PaO_2_/FiO_2_ = ratio of partial pressure of arterial oxygen to faction of inspired oxygen concentration; RR = respiratory rate; VTave = average tidal volume; P_plat_ = plateau pressure; PTP = pressure time product; mean Paw, = mean airway pressure; peak P_L_, = peak transpulmonary pressure; mean P_L_, = mean transpulmonary pressure; Peso = esophageal pressure; Pgas = intragastric pressure; ΔPes = change of esophageal pressure, PTP = pressure time product

*p < 0.05,BIPAP_AP_ vs. BIPAP_SB_ group at the same time.

**Fig 4:**
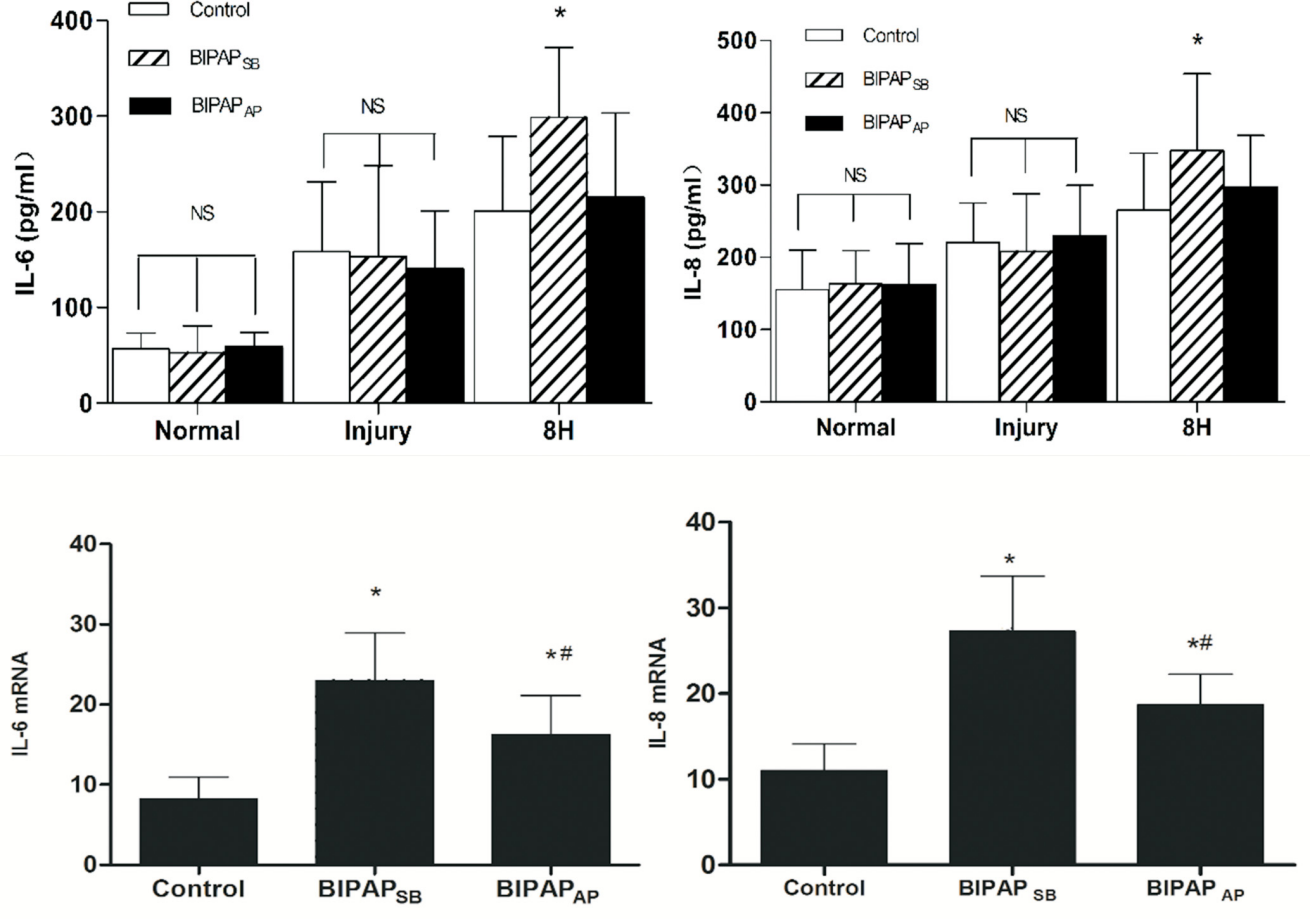
The Levels of IL-6 and IL-8 in plasma and the mRNA expression levels in the lung tissue. The Levels of interleukin (IL)-6 and IL-8 in plasma (*P < 0.05 vs. other groups) and the messenger RNA (mRNA) expression levels of IL-6 and IL-8 in the lung tissue after 8h mechanical ventilation (*P < 0.05 vs. control group; #P < 0.05 vs. BIPAP_AP_ group;). BIPAP_SB_ = biphasic positive airway pressure with SB, n = 6; BIPAP_AP_ = biphasic positive airway pressure with abdominal muscles paralysis, n = 6; SB = spontaneous breathing; NS = no significantly difference;

As shown in Fig 4: BIPAP_AP_ group yielded lower levels of IL-6 (216.6±48.0 pg/ml) and IL-8 (246.8±78.2 pg/ml) in plasma compared with BIPAP_SB_ groups (IL-6:297.5±71.2 pg/ml;IL-8:357.5±69.3 pg/ml) after 8h MV (*p*<0.05).Moreover, mRNA expression levels of IL-6 and IL-8 in lung tissue were lower in BIPAP_AP_ group (IL-6:15.0±3.8, IL-8: 18.9±6.8) than in BIPAP_SB_ group (IL-6:21.2±3.7, IL-8:29.5±7.9), and all experimental groups were higher than the control group.

**Fig 5:**
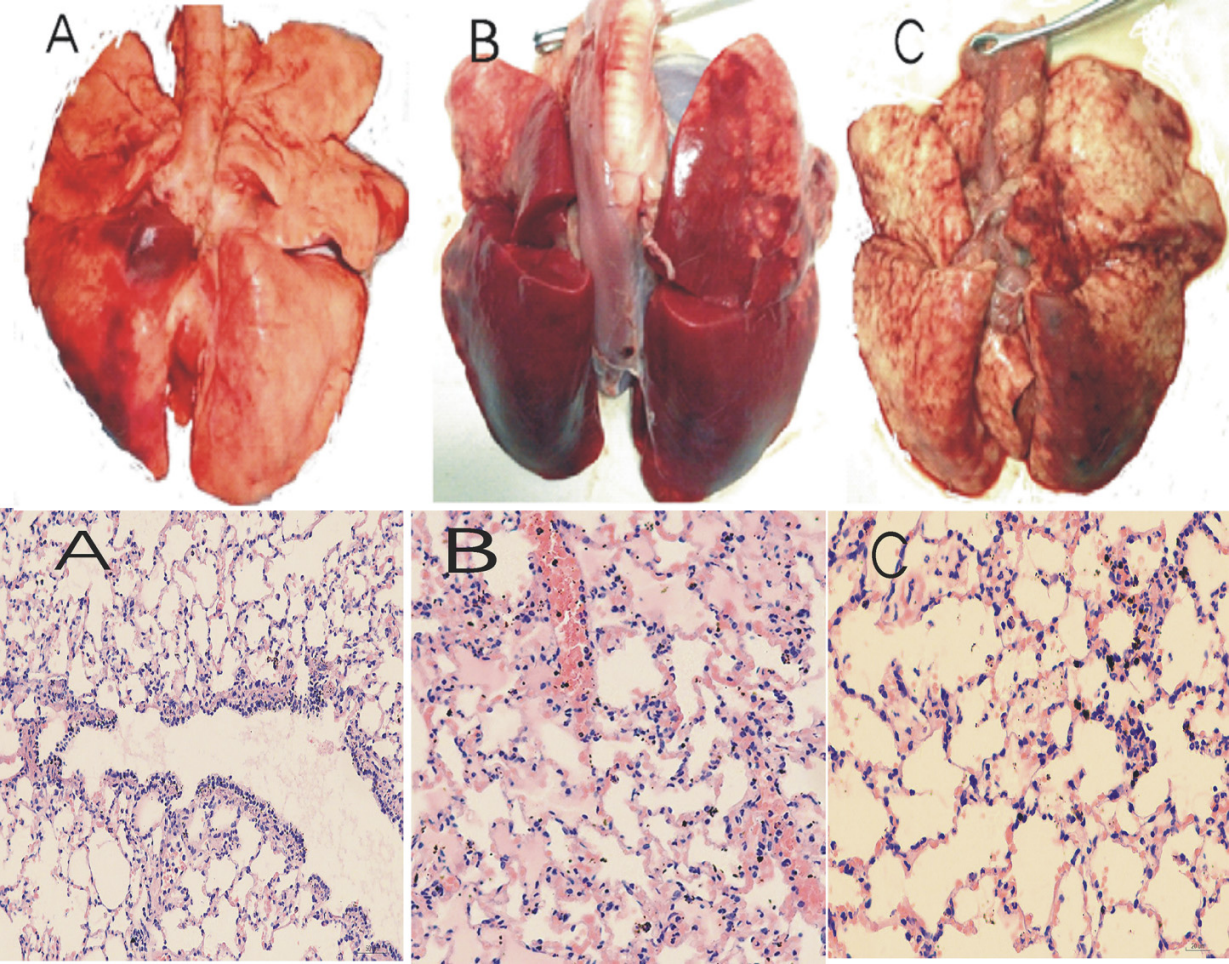
Histopathological examination. Representative appearances and photomicrographs of hematoxylineosin–stained lung sections (magnification ×200) from control group (A, n = 6), BIPAP_SB_ = biphasic positive airway pressure with SB (B, n = 6), and BIPAP_AP_ = biphasic positive airway pressure with abdominal muscles paralysis (C, n = 6).The control group had minimal alveolar congestion, and inflammatory cell infiltration. The BIPAP_AP_ group showed mild thickening of the alveolar walls, alveolar congestion, and hemorrhage. In the BIPAP_SB_ group, inflammatory cell infiltration, thickening of the alveolar walls, alveolar congestion, and more prominent hemorrhagic areas were observed.

As shown in Table 2, The sum of lung injury scores was lower in BIPAP_AP_ group (22.5±2.0) than that in BIPAP_SB_ group (25.2±2.1), but the sum of scores in the experimental groups was higher than that in the control group. The BIPAP_AP_ group showed less congestion, alveolar edema, alveolar infiltration of neutrophils and interstitial, and less infiltration of lymphocyte. The BIPAP_SB_ group showed increased alveolar collapse, alveolar congestion, infiltration of inflammatory cells, and interstitial edema with hyaline membrane formation (Fig 5).

**Table 2:** Histological subscores in experimental groups.

	BIPAP_AP_	BIPAP_SB_	*P* value
**Congestion**	2.1±0.4	2.8±0.5	0.009
**Edema, interstitial**	2.2±0.8	2.4±0.5	0.316
**Edema, alveolar**	2.2±0.5	2.7±0.7	0.007
**Granulocyte infiltrate, interstitial**	2.2±0.5	2.4±0.6	0.256
**Granulocyte infiltrate, alveolar**	2.1±0.6	2.5±0.6	0.042
**Erythrocyte infiltrate, interstitial**	2.4±0.6	2.5±0.4	0.704
**Erythrocyte infiltrate, alveolar**	2.4±0.8	2.6±0.6	0.366
**Lymphocyte infiltrate, interstitial**	2.3±0.7	2.7±0.5	0.017
**Microthrombi**	2.2±0.9	2.3±0.3	0.255
**Fibrinous exudate, interstitia**	2.3±0.5	2.4±0.5	0.662
**Fibrinous exudate, alveolar**	2.3±0.4	2.2±0.3	0.714
**Cumulative score**	**22.5±2.0**	**25.2±2.1**	***P*<0.01**

Values are means ± SD.BIPAP_SB_ = biphasic positive airway pressure with SB; BIPAP_AP_ = biphasic positive airway pressure with abdominal muscles paralysis; SB = spontaneous breathing; Grading as: 0, minimal changes; 1, mild; 2, moderate; 3, severe; 4, maximal changes

## Discussion

In an oleic acid- induced model of experimental ARDS in beagles, our findings suggested that abdominal muscle activity during mechanically ventilation increases lung injury in severe acute respiratory distress syndrome.

To our knowledge, no previous experimental study has investigated the effect of abdominal muscle on lung damage in ARDS. We used an oleic acid-induced experimental ARDS because this model has reproduced many basic characteristics of ARDS [17]. For different animals, the same dose of oleic acid administered by the same route can induce a reasonably reproducible lung injury [24]. Different ventilated strategies have been proposed in ARDS. BIPAP mode was chosen because it can be easily modulated to maintain comparable levels of ventilator support in the three groups. By the methods of adjusting ventilator setting, the levels of mean Paw were comparable, the only difference was with or without abdominal muscles activity which were visible from electromyogram. In our study, we used a super syringe method to make a static pressure–volume curve and our results showed that the lower inflection points were 8-9cm H_2_O for lung injury. So we set P_low_ (PEEP) at 10 cm H_2_O for the three groups.

### Gas Exchange

With comparable ventilator setting, the oxygenation index were significantly higher in BIPAP_AP_ group than BIPAP_SB_ group after 2h MV. This outcome proved that abdominal muscle activity may worsen gas exchange. There were several reasons to explain this phenomenon: first, activity of the abdominal muscle was associated with decreased EELV. Douglas and colleagues [25] observed in their study that EELV was parallel to oxygenation. Similarly, a better gas exchange was also observed after abdominal muscle paralysis. Second, activity of the abdominal muscle was associated with increased PTP, which represented a decrease of total work of breathing and oxygen consumption.Third, activity of the abdominal muscle resulted in an decreased P_L_ on P_high_, which could recruit the collapse alveolar units and result in greater lung units available for oxygenation [26]. Fourth, abdominal muscle activity increase of intra-abdominal pressure which is associated with the decrease of P_L_ and respiratory compliance on T_high_, which can lead to a loss in lung volume and hypoxemia episodes [27]. Fifth, activity of the abdominal muscle resulted in worsen blood reperfusion to nondependent lung area, and led to a higher VD/VT and greater inhomogeneity of lung ventilation to perfusion. All the above factors can worsen gas exchange.

### Lung Injury

Ventilator-associated lung injury (VALI) includes volutrauma, atelectrauma and biotrauma., and these injuries may eventually lead to severe systemic inflammatory response and multiple organ failure. No previous studies have proved the relationships between abdominal muscle and VALI in ARDS. In an oleic acid-induced ARDS model, our study showed that BIPAP_AP_ had lower mRNA expression of IL-6 and IL-8 in lung tissues and less total cumulative histopathological lung injury scores compared with BIPAP_SB_ group. These findings suggested that activity of the abdominal muscle during mechanically ventilation was one of the injurious factors in severe ARDS. Various mechanisms may explain the findings:①Activity of the abdominal muscle can increase the value of △Pes which has been shown to promote the formation of pulmonary edema and aggravate lung injury[26]. ②Activity of the abdominal muscle can increase end-expiratory alveolar pressure at the start of expiratory. ③Activity of the abdominal muscle can increase intra-abdominal hypertension. It has been proved that activity of the abdominal muscles can increase IAP by up to 20 cmH_2_O [28]. When intra-abdominal hypertension existed, unopposed increase of intra-abdominal pressure by spontaneous expiratory can cause greater lung injury by reducing P_L_ in dependent zones [13]. ④Activity of the abdominal muscle can decrease EELV, and make part of lung units breathing in the lower inflection point of the P-V curve and cause so-called “low volume injury” which is associated with the cyclic opening–closing of lung units and aggregates lung injury by interfacial forces. ⑤Activity of the abdominal muscle allows PEEP to be bad controlled and resulting in increased “atelectrauma”. ⑥Activity of the abdominal muscle resulted in decreased P_L_ which was presumed to aggravate lung injury. ⑦Activity of the abdominal muscle resulted in decreased EELV, so lung strain, a major determinant of VALI, might be further increased.

## Limitations

There are several limitations in this study. First, the work was done in an oleic acid-induced ARDS model. Therefore, we are not sure whether these results can be reproduced in the other ARDS models. Second, due to protective strategy with a LTV used in this experiment, we cannot preclude the protective effects of abdominal muscle on a high tidal volume injurious ventilation. Third, the RR and nervous distribution of canine may not be the same as human being, so we cannot guarantee our data can be used for patients and further studies are needed. Finally, in BIPAP_AP_ group, Lidocaine and Ropivacaine hydrochloride were used for epidural anesthesia to paralyze abdominal muscles, so we cannot rule out the possibility that these drugs affected pulmonary inflammatory response.

## Conclusion

In conclusion, in a canine model of oleic acid-induced severe ARDS, abdominal muscle activity during mechanically ventilation increases lung injury in severe acute respiratory distress syndrome, so abdominal muscles paralysis minimize ventilator-induced lung injury in early, severe patients with ARDS.

## Supporting Information

S1 Fig Representative respiratory tracings of BIPAP_SB_ and BIPAP_AP_ group in representative animals. (PDF)

S2 Fig Representative appearance of the lung after 8h ventilation in different groups. (PDF)

S1 Table Representative data in different groups. (XLSX)
