# Correction: Abdominal Muscle Activity during Mechanical Ventilation Increases Lung Injury in Severe Acute Respiratory Distress Syndrome

**DOI:** 10.1371/journal.pone.0149325

**Published:** 2016-02-09

**Authors:** Xianming Zhang, Weiliang Wu, Yongcheng Zhu, Ying Jiang, Juan Du, Rongchang Chen

There is an error in affiliation 1 for authors Xianming Zhang and Juan Du. Affiliation 1 should be: Department of Respiratory Medicine, First Affiliated Hospital of Guizhou Medical University, Guizhou, China
