# Supplementary material for: Abdominal Muscle Activity during Mechanical Ventilation Increases Lung Injury in Severe Acute Respiratory Distress Syndrome
Source: PLoS One. 2016 Jan 8;11(1):e0145694. doi: 10.1371/journal.pone.0145694 (PMC4712828; doi:10.1371/journal.pone.0145694)
Supplement: S1 Fig — (PDF) [file pone.0145694.s001.pdf]

BIPAP$_{SB}$

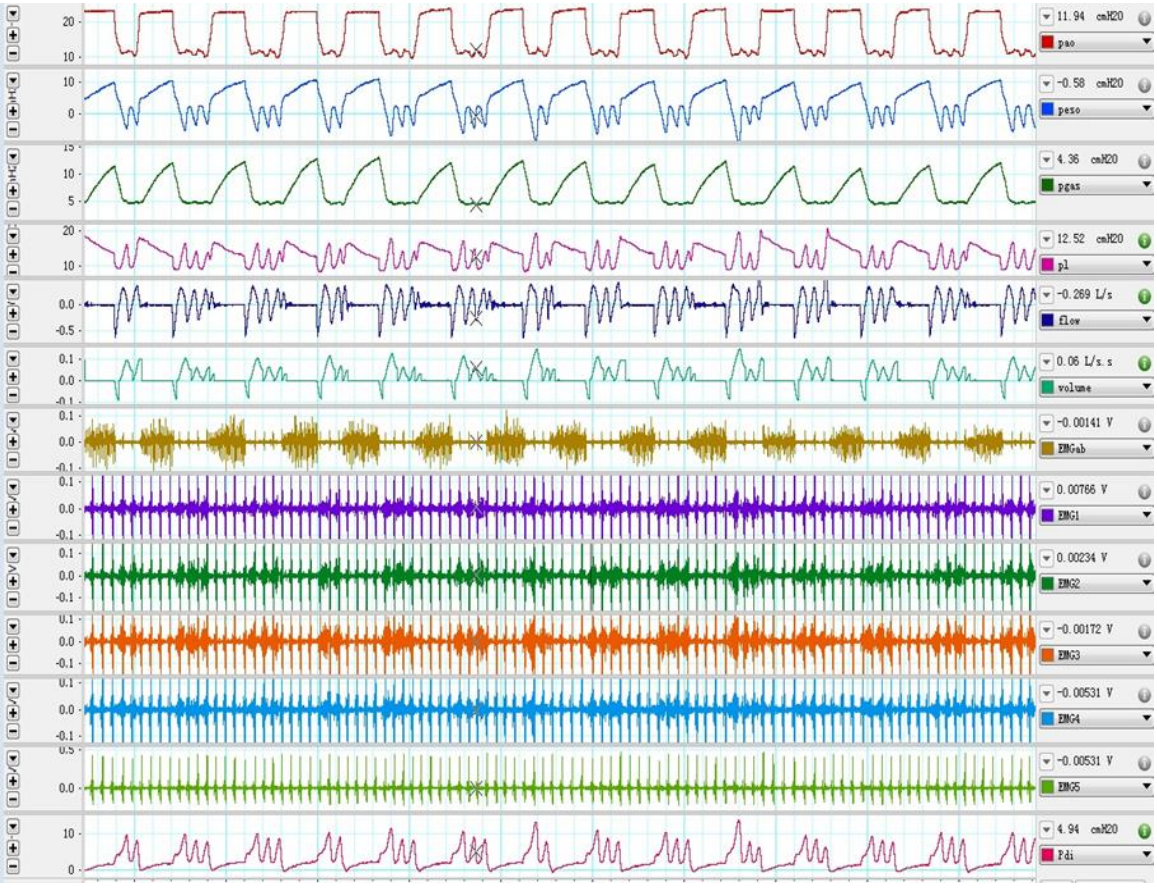

Representative respiratory tracings of airway pressure(Paw), esophageal pressure (Pes) 、intragastric pressure （Pgas）, transpumonary pressure（PL）, Airflow，tidal volume，abdominal muscles surface electromyography （ EMGab ） and diaphragmatic esophageal surface electromyography（EMGdi) in BIPAP$_{SB}$ group in representative animals.

BIPAP$_{AP}$

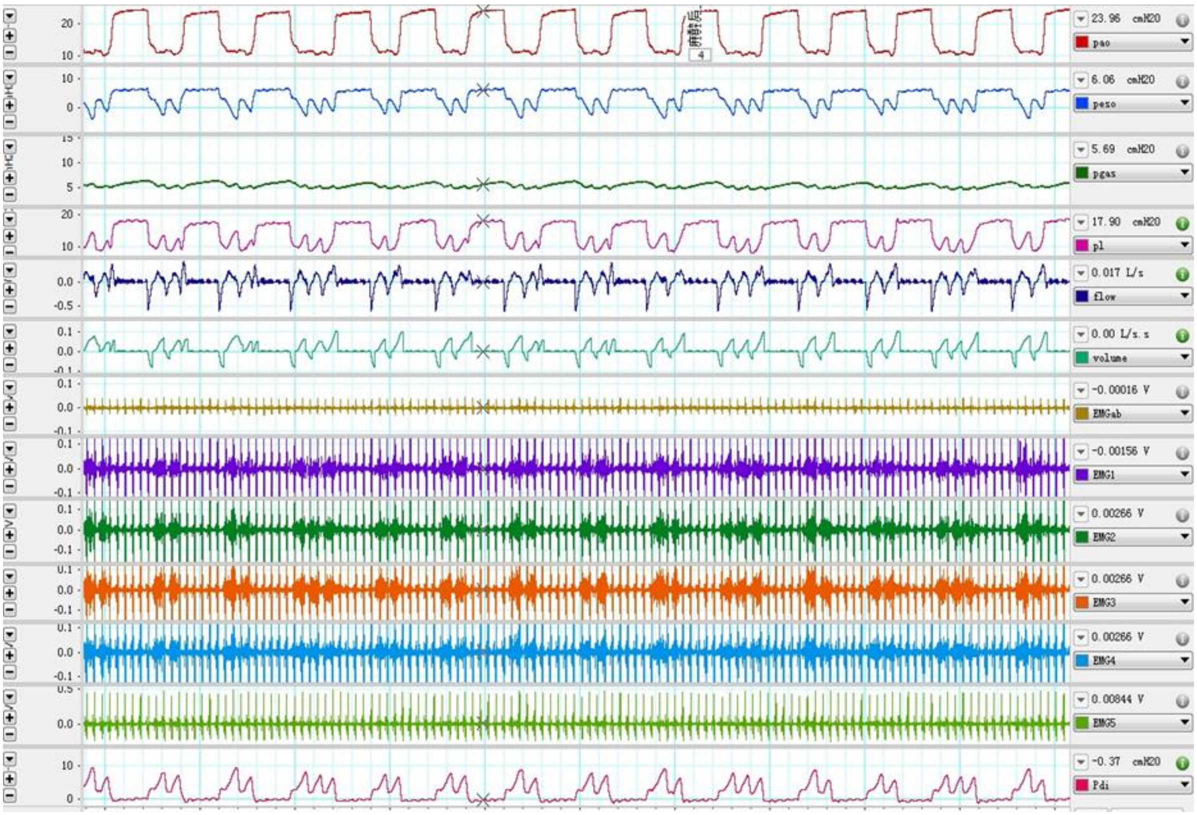

Representative respiratory tracings of airway pressure(Paw), esophageal pressure (Pes) 、 intragastric pressure （Pgas），transpumonary pressure（PL），Airflow，tidal volume，abdominal muscles surface electromyography（EMGab），diaphragmatic esophageal surface electromyography （EMGdi)（1-5）and transdiaphragmatic pressure（Pdi） in BIPAP$_{AP}$ group in representative animals
