# Supplementary material for: Abdominal Muscle Activity during Mechanical Ventilation Increases Lung Injury in Severe Acute Respiratory Distress Syndrome
Source: PLoS One. 2016 Jan 8;11(1):e0145694. doi: 10.1371/journal.pone.0145694 (PMC4712828; doi:10.1371/journal.pone.0145694)
Supplement: S2 Fig — (PDF) [file pone.0145694.s002.pdf]

Figure S2 Representative Appearance of the lung after 8h ventilation in different groups

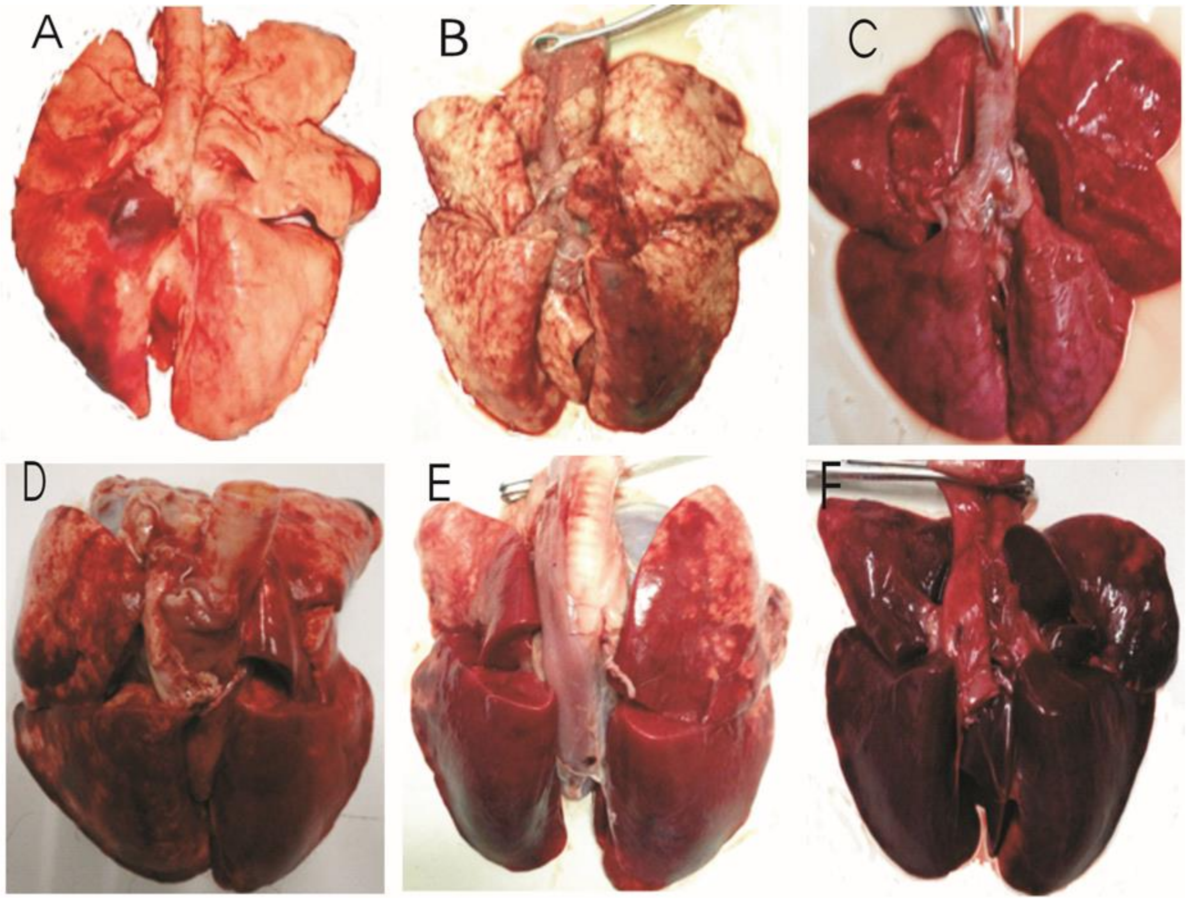

Control group: figure A; $BIPAP_{AP}$ group:figure B and C ,$BIPAP_{SB}$ group: figure ,D,E,F
